# 1,2-Bis(2-nitro­phen­yl)disulfane

**DOI:** 10.1107/S1600536809042780

**Published:** 2009-10-23

**Authors:** Mingzhi Song, Chuangang Fan

**Affiliations:** a College of Chemistry and Chemical Technology, Binzhou University, Binzhou 256600, Shandong, People’s Republic of China; b College of Chemistry and Chemical Engineering, China University of Petroleum, Qingdao Shandong, 266555, People’s Republic of China

## Abstract

In the title compound, C_12_H_8_N_2_O_4_S_2_, the dihedral angle between the two benzene rings is 67.82 (9)°. In the crystal, weak inter­molecular C—H⋯O hydrogen bonds link the mol­ecules.

## Related literature

For background to disulfides, see: Kitamura *et al.* (1991 ▶); Palmer *et al.* (1995 ▶); Ramadas & Srinivasan (1995 ▶). For related structures, see: Glidewell *et al.* (2000 ▶);
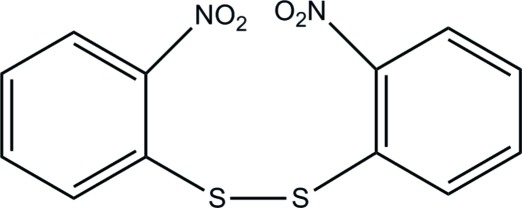


## Experimental

### 

#### Crystal data

C_12_H_8_N_2_O_4_S_2_
*M* *_r_* = 308.32
Monoclinic, 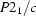

*a* = 8.3762 (9) Å
*b* = 21.028 (2) Å
*c* = 8.1011 (10) Å
β = 111.768 (1)°
*V* = 1325.1 (3) Å^3^
*Z* = 4
Mo *K*α radiation
μ = 0.42 mm^−1^
*T* = 298 K
0.44 × 0.18 × 0.13 mm

#### Data collection

Bruker SMART APEX CCD area-detector diffractometer
Absorption correction: multi-scan (*SADABS*; Sheldrick, 1996 ▶) *T*_min_ = 0.838, *T*_max_ = 0.948
6598 measured reflections
2317 independent reflections
1507 reflections with *I* > 2σ(*I*)
*R*_int_ = 0.045

#### Refinement

*R*[*F* ^2^ > 2σ(*F* ^2^)] = 0.046
*wR*(*F* ^2^) = 0.117
*S* = 0.92
2317 reflections
181 parameters
H-atom parameters constrained
Δρ_max_ = 0.32 e Å^−3^
Δρ_min_ = −0.17 e Å^−3^

### 

Data collection: *SMART* (Siemens, 1996 ▶); cell refinement: *SAINT* (Siemens, 1996 ▶); data reduction: *SAINT*; program(s) used to solve structure: *SHELXS97* (Sheldrick, 2008 ▶); program(s) used to refine structure: *SHELXL97* (Sheldrick, 2008 ▶); molecular graphics: *SHELXTL* (Sheldrick, 2008 ▶); software used to prepare material for publication: *SHELXTL*.

## Supplementary Material

Crystal structure: contains datablocks I, global. DOI: 10.1107/S1600536809042780/bq2169sup1.cif

Structure factors: contains datablocks I. DOI: 10.1107/S1600536809042780/bq2169Isup2.hkl

Additional supplementary materials:  crystallographic information; 3D view; checkCIF report

## Figures and Tables

**Table 1:** Hydrogen-bond geometry (Å, °)

*D*—H⋯*A*	*D*—H	H⋯*A*	*D*⋯*A*	*D*—H⋯*A*
C6—H6⋯O4^i^	0.93	2.53	3.231 (4)	133
C11—H11⋯O3^ii^	0.93	2.54	3.293 (4)	138

Symmetry codes: (i) 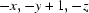; (ii) 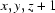.

**Fig. 1.**
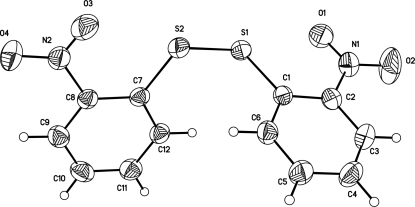
A view of (I) showing the atomic numbering scheme and 30% probability displacement ellipsoids.

**Fig. 2.**
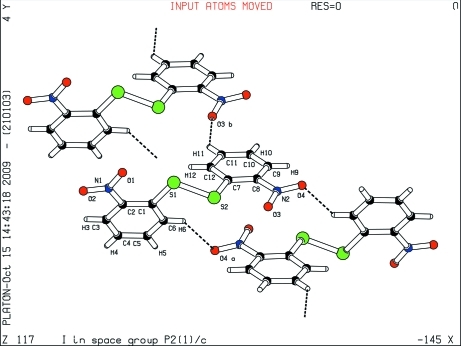
A partial packing view of (I) showing the weak C-H..O hydrogen bonds with dashed lines. Symmetry codes: (a) -x, -y+1, -z; (b) x, y, z+1.

## supplementary crystallographic information

### Comment

Disulfides form an important class of compounds with respect to their synthetic and industrial applications and biological occurrence (Palmer, *et al.*, 1995). Their syntheses remain an active area of interest in organic chemistry (Kitamura, *et al.*, 1991). Disulfides are used as sulphenylating agents for enolates and other anions industrially; they find a wide range of applications as vulcanizing agents for rubber and elastomers. Several classes of naturally occurring compounds contain disulfides including gliotoxin and lipoic acid (Ramadas, *et al.*, 1995).

In the title compound (I), (Fig. 1), the bond lengths an angles are normal and are comparable to the values observed in similar compounds (Glidewell,*et al.*, 2000).

In the crystal structure, the S—S bond length in the molecule is 2.0584 (12)° (S1—S2), showing the single bond character. Meanwhile, the dihedral angle between the benzene rings (C1-C6) and (C7-C12) is 67.82 (9)°, indicating that the two aromatic ring planes are not coplanar.

Moreover, the crystal supramolecular structure was built from the connections of intermolecular weak C-H···O hydrogen bonds interactions (Fig. 2).

### Experimental

*o*-Nitrochlorobenzene (10.0 mmol), 20 ml ethanol and sodium disulfide (12.0 mmol) were mixed in 50 ml flash. After refluxing 6 h, the resulting mixture was cooled to room temperature, and recrystalized from ethanol, and afforded the title compound as a crystalline solid. Elemental analysis: calculated for C_12_H_8_N_2_O_4_S_2_: C 46.74, H 2.62, N 9.09%; found: C 46.65, H 2.66, N 9.14%.

### Refinement

All H atoms were placed in geometrically idealized positions (C—H 0.93 Å) and treated as riding on their parent atoms, with *U*_iso_(H) = 1.2*U*_eq_(C).

### Crystal data

**Table:**

C_12_H_8_N_2_O_4_S_2_	*F*(000) = 632
*M**_r_* = 308.32	*D*_x_ = 1.545 Mg m^−^^3^
Monoclinic, *P*2_1_/*c*	Mo *K*α radiation, λ = 0.71073 Å
Hall symbol: -P 2ybc	Cell parameters from 1601 reflections
*a* = 8.3762 (9) Å	θ = 2.6–24.7°
*b* = 21.028 (2) Å	µ = 0.42 mm^−^^1^
*c* = 8.1011 (10) Å	*T* = 298 K
β = 111.768 (1)°	Needle, yellow
*V* = 1325.1 (3) Å^3^	0.44 × 0.18 × 0.13 mm
*Z* = 4	

### Data collection

**Table:**

Bruker SMART APEX CCD area-detector diffractometer	2317 independent reflections
Radiation source: fine-focus sealed tube	1507 reflections with *I* > 2σ(*I*)
graphite	*R*_int_ = 0.045
phi and ω scans	θ_max_ = 25.0°, θ_min_ = 1.9°
Absorption correction: multi-scan (*SADABS*; Sheldrick, 1996)	*h* = −8→9
*T*_min_ = 0.838, *T*_max_ = 0.948	*k* = −25→20
6598 measured reflections	*l* = −9→9

### Refinement

**Table:**

Refinement on *F*^2^	Primary atom site location: structure-invariant direct methods
Least-squares matrix: full	Secondary atom site location: difference Fourier map
*R*[*F*^2^ > 2σ(*F*^2^)] = 0.046	Hydrogen site location: inferred from neighbouring sites
*wR*(*F*^2^) = 0.117	H-atom parameters constrained
*S* = 0.92	*w* = 1/[σ^2^(*F*_o_^2^) + (0.0627*P*)^2^] where *P* = (*F*_o_^2^ + 2*F*_c_^2^)/3
2317 reflections	(Δ/σ)_max_ < 0.001
181 parameters	Δρ_max_ = 0.32 e Å^−^^3^
0 restraints	Δρ_min_ = −0.16 e Å^−^^3^

### Special details

**Table:**

Geometry. All e.s.d.'s (except the e.s.d. in the dihedral angle between two l.s. planes) are estimated using the full covariance matrix. The cell e.s.d.'s are taken into account individually in the estimation of e.s.d.'s in distances, angles and torsion angles; correlations between e.s.d.'s in cell parameters are only used when they are defined by crystal symmetry. An approximate (isotropic) treatment of cell e.s.d.'s is used for estimating e.s.d.'s involving l.s. planes.
Refinement. Refinement of *F*^2^ against ALL reflections. The weighted *R*-factor *wR* and goodness of fit *S* are based on *F*^2^, conventional *R*-factors *R* are based on *F*, with *F* set to zero for negative *F*^2^. The threshold expression of *F*^2^ > σ(*F*^2^) is used only for calculating *R*-factors(gt) *etc*. and is not relevant to the choice of reflections for refinement. *R*-factors based on *F*^2^ are statistically about twice as large as those based on *F*, and *R*- factors based on ALL data will be even larger.

### Fractional atomic coordinates and isotropic or equivalent isotropic displacement parameters (Å^2^)

**Table:**

	*x*	*y*	*z*	*U*_iso_*/*U*_eq_	
S1	0.09105 (9)	0.26719 (4)	0.07988 (11)	0.0597 (3)	
S2	0.01195 (10)	0.35209 (4)	−0.05162 (11)	0.0621 (3)	
N1	0.3638 (4)	0.16520 (13)	0.2840 (4)	0.0692 (8)	
N2	−0.2348 (3)	0.46767 (13)	−0.1758 (4)	0.0619 (7)	
O1	0.2185 (3)	0.15373 (11)	0.1813 (4)	0.0830 (7)	
O2	0.4683 (4)	0.12365 (13)	0.3492 (5)	0.1297 (13)	
O3	−0.1569 (3)	0.44411 (12)	−0.2595 (3)	0.0852 (7)	
O4	−0.3526 (4)	0.50494 (12)	−0.2385 (4)	0.0995 (9)	
C1	0.3037 (3)	0.28110 (13)	0.2387 (4)	0.0473 (7)	
C2	0.4143 (4)	0.23134 (13)	0.3261 (4)	0.0531 (8)	
C3	0.5754 (4)	0.24239 (16)	0.4559 (5)	0.0685 (9)	
H3	0.6454	0.2084	0.5124	0.082*	
C4	0.6305 (4)	0.30339 (18)	0.5001 (5)	0.0779 (10)	
H4	0.7382	0.3112	0.5869	0.093*	
C5	0.5255 (4)	0.35347 (16)	0.4151 (5)	0.0719 (10)	
H5	0.5631	0.3950	0.4448	0.086*	
C6	0.3655 (4)	0.34241 (14)	0.2866 (4)	0.0594 (8)	
H6	0.2972	0.3768	0.2305	0.071*	
C7	−0.0762 (3)	0.39748 (13)	0.0814 (4)	0.0485 (7)	
C8	−0.1831 (3)	0.44971 (13)	0.0136 (4)	0.0494 (7)	
C9	−0.2478 (4)	0.48675 (15)	0.1161 (5)	0.0633 (9)	
H9	−0.3200	0.5209	0.0660	0.076*	
C10	−0.2036 (4)	0.47214 (17)	0.2937 (5)	0.0714 (10)	
H10	−0.2442	0.4969	0.3649	0.086*	
C11	−0.0996 (4)	0.42093 (16)	0.3642 (4)	0.0671 (9)	
H11	−0.0699	0.4113	0.4839	0.081*	
C12	−0.0371 (3)	0.38288 (15)	0.2607 (4)	0.0550 (8)	
H12	0.0307	0.3477	0.3109	0.066*	

### Atomic displacement parameters (Å^2^)

**Table:**

	*U*^11^	*U*^22^	*U*^33^	*U*^12^	*U*^13^	*U*^23^
S1	0.0511 (4)	0.0572 (5)	0.0614 (6)	−0.0011 (4)	0.0099 (4)	−0.0114 (4)
S2	0.0619 (5)	0.0762 (6)	0.0455 (5)	0.0124 (4)	0.0167 (4)	−0.0020 (4)
N1	0.0631 (18)	0.0618 (18)	0.083 (2)	0.0056 (16)	0.0281 (17)	0.0059 (16)
N2	0.0582 (16)	0.0521 (17)	0.064 (2)	−0.0038 (13)	0.0096 (15)	0.0000 (15)
O1	0.0839 (18)	0.0626 (16)	0.093 (2)	−0.0072 (13)	0.0213 (16)	−0.0079 (13)
O2	0.097 (2)	0.0610 (17)	0.201 (4)	0.0235 (16)	0.020 (2)	0.026 (2)
O3	0.0949 (18)	0.106 (2)	0.0536 (15)	0.0135 (16)	0.0258 (14)	0.0114 (14)
O4	0.111 (2)	0.0818 (18)	0.086 (2)	0.0278 (17)	0.0132 (16)	0.0264 (15)
C1	0.0420 (15)	0.0549 (18)	0.0471 (18)	0.0003 (14)	0.0192 (13)	−0.0036 (14)
C2	0.0537 (17)	0.0514 (18)	0.059 (2)	0.0004 (14)	0.0272 (16)	0.0023 (15)
C3	0.0526 (18)	0.075 (2)	0.071 (2)	0.0103 (17)	0.0154 (17)	0.0117 (19)
C4	0.0493 (19)	0.087 (3)	0.082 (3)	−0.0052 (19)	0.0056 (18)	−0.003 (2)
C5	0.0541 (19)	0.068 (2)	0.084 (3)	−0.0084 (18)	0.0146 (18)	−0.009 (2)
C6	0.0504 (17)	0.0541 (19)	0.071 (2)	0.0010 (15)	0.0194 (16)	−0.0010 (16)
C7	0.0401 (14)	0.0565 (18)	0.0460 (18)	−0.0034 (13)	0.0126 (13)	−0.0048 (14)
C8	0.0443 (15)	0.0472 (17)	0.0504 (19)	−0.0073 (13)	0.0103 (14)	−0.0015 (14)
C9	0.0589 (19)	0.056 (2)	0.072 (3)	−0.0018 (15)	0.0210 (18)	−0.0066 (17)
C10	0.072 (2)	0.075 (2)	0.073 (3)	0.0013 (19)	0.034 (2)	−0.021 (2)
C11	0.066 (2)	0.085 (2)	0.053 (2)	0.0054 (19)	0.0263 (17)	−0.0023 (18)
C12	0.0516 (17)	0.066 (2)	0.0468 (19)	0.0048 (15)	0.0179 (15)	0.0018 (15)

### Geometric parameters (Å, °)

**Table:**

S1—C1	1.793 (3)	C4—H4	0.9300
S1—S2	2.0584 (12)	C5—C6	1.378 (4)
S2—C7	1.791 (3)	C5—H5	0.9300
N1—O2	1.210 (3)	C6—H6	0.9300
N1—O1	1.217 (3)	C7—C8	1.395 (4)
N1—C2	1.457 (4)	C7—C12	1.399 (4)
N2—O3	1.208 (3)	C8—C9	1.388 (4)
N2—O4	1.214 (3)	C9—C10	1.380 (5)
N2—C8	1.480 (4)	C9—H9	0.9300
C1—C6	1.390 (4)	C10—C11	1.370 (5)
C1—C2	1.403 (4)	C10—H10	0.9300
C2—C3	1.389 (4)	C11—C12	1.394 (4)
C3—C4	1.365 (4)	C11—H11	0.9300
C3—H3	0.9300	C12—H12	0.9300
C4—C5	1.381 (4)		
			
C1—S1—S2	105.87 (10)	C4—C5—H5	119.7
C7—S2—S1	106.03 (11)	C5—C6—C1	121.6 (3)
O2—N1—O1	122.2 (3)	C5—C6—H6	119.2
O2—N1—C2	119.1 (3)	C1—C6—H6	119.2
O1—N1—C2	118.6 (3)	C8—C7—C12	116.8 (3)
O3—N2—O4	123.7 (3)	C8—C7—S2	122.0 (2)
O3—N2—C8	117.7 (3)	C12—C7—S2	121.2 (2)
O4—N2—C8	118.6 (3)	C9—C8—C7	122.8 (3)
C6—C1—C2	116.3 (3)	C9—C8—N2	116.6 (3)
C6—C1—S1	121.3 (2)	C7—C8—N2	120.5 (3)
C2—C1—S1	122.3 (2)	C10—C9—C8	119.0 (3)
C3—C2—C1	122.1 (3)	C10—C9—H9	120.5
C3—C2—N1	116.9 (3)	C8—C9—H9	120.5
C1—C2—N1	120.9 (3)	C11—C10—C9	119.5 (3)
C4—C3—C2	119.7 (3)	C11—C10—H10	120.2
C4—C3—H3	120.2	C9—C10—H10	120.2
C2—C3—H3	120.2	C10—C11—C12	121.6 (3)
C3—C4—C5	119.6 (3)	C10—C11—H11	119.2
C3—C4—H4	120.2	C12—C11—H11	119.2
C5—C4—H4	120.2	C11—C12—C7	120.2 (3)
C6—C5—C4	120.6 (3)	C11—C12—H12	119.9
C6—C5—H5	119.7	C7—C12—H12	119.9

### Hydrogen-bond geometry (Å, °)

**Table:**

*D*—H···*A*	*D*—H	H···*A*	*D*···*A*	*D*—H···*A*
C6—H6···O4^i^	0.93	2.53	3.231 (4)	133
C11—H11···O3^ii^	0.93	2.54	3.293 (4)	138

Symmetry codes: (i) −*x*, −*y*+1, −*z*; (ii) *x*, *y*, *z*+1.
